# A cycling and education intervention for the treatment of hip osteoarthritis: A quality improvement replication programme

**DOI:** 10.1177/2050312120946522

**Published:** 2020-07-31

**Authors:** Thomas W Wainwright, Louise C Burgess, Tikki Immins, Neil Cowan, Robert G Middleton

**Affiliations:** 1 Orthopaedic Research Institute, Bournemouth University, Bournemouth, UK; 2 The Royal Bournemouth and Christchurch Hospitals NHS Trust, Bournemouth, UK; 3 Royal Perth Bentley Group, Perth, WA, Australia

**Keywords:** Hip osteoarthritis, exercise therapy, patient education, self-management, group exercise, cycling

## Abstract

**Objectives:**

The Cycling against Hip Pain programme is a 6-week exercise and education treatment pathway for people with hip osteoarthritis. Preliminary results of the Cycling against Hip Pain programme found significant improvements in clinical and patient-reported outcome measures for patients referred from primary care. This article evaluates the effectiveness of the changes made to the pathway in a quality improvement replication programme.

**Methods:**

The replicated Cycling against Hip Pain programme was delivered between February 2018 and September 2019 in a region of England with a high percentage of adults aged over 65 years. All participants were referred from the orthopaedic outpatient department of the funding hospital (secondary care). The programme was delivered at a local leisure centre and combined 30 min of education on osteoarthritis with 30 min of progressive static cycling, once a week for 6 weeks.

**Results:**

The participants on the replicated Cycling against Hip Pain programme did not differ from the original cohort in terms of age or pre-programme weight, however, presented with worse hip symptoms at baseline. Consistent with the findings from the original cohort, participants demonstrated significant improvements to their Oxford Hip Score, 30-s chair stand performance, Timed Up and Go score, Hip Osteoarthritis Outcome Score function and pain, EQ5D health rating, EQ5D-5L score and pain at rest and on weight bearing. In addition, participants reported an increase in knowledge, confidence and motivation to exercise.

**Conclusion:**

A 6-week cycling and education intervention for the treatment of hip osteoarthritis provided benefits to function, pain and quality of life for patients referred from secondary care. These results are consistent with findings from patients who were referred from primary care and further support the potential of the pathway in the conservative management of hip osteoarthritis.

## Introduction

Osteoarthritis is a common musculoskeletal condition within older adults, with an estimated 33% of people aged 45 and over (8.75 million) having sought treatment for the disease in the United Kingdom.^1^ Almost two and a half million adults (10.9%) aged over 45 have osteoarthritis of the hip, which can cause debilitating pain leading to referral for total hip replacement surgery.^2^ While this procedure is clinically effective^3–5^ and cost-efficient,^6^ surgery presents major risks and complications such as dislocation, blood clots and infection.^7^ Encouraging patients to self-manage their symptoms in the earlier stages of the disease process may have a longer term benefit.

The National Institute for Health and Care Excellence (NICE) guidelines,^8^ in line with other international recommendations,^9^ suggest a combination of education and self-management, exercise (aerobic and local muscle strengthening) and weight loss where appropriate as conservative treatment for osteoarthritis. The research evidence summarising the benefits of exercise for the self-management of hip osteoarthritis is expanding;^10–13^ however, the optimal form and dose of exercise has not yet been elucidated.^11,14^

## The local problem

The Cycling against Hip Pain (CHAIN) programme was conceived in 2014 and aims to implement the NICE guidelines for treatment of hip osteoarthritis through a 6-week cycling and education intervention.^15^ The programme was first delivered as a feasibility project in a region of the United Kingdom with a high percentage of adults aged over 65 years.^16^ Indeed, between April 2016 and March 2019, 2,325 patients underwent total hip replacement surgery at the local National Health Service (NHS) hospital, over three times the national average of 717 procedures for this time period.^17^ The short-term results of the CHAIN programme (*n* = 96) found statistically significant improvements in clinical and patient-reported outcome measures.^15^ In addition, participants reported psychological benefits, including increased confidence in managing their own hip pain and an increase in motivation to exercise.^15^ A mid-term evaluation of this cohort found that at 5-year follow-up, 100% of participants were still active and 57% had avoided surgical intervention for the treatment of their hip pain.^18^

The CHAIN programme was originally delivered with seedcorn funding and funding in kind from a range of stakeholders, including primary and secondary care. Following completion of the initial feasibility programme, the local NHS trust (The Royal Bournemouth and Christchurch Hospitals NHS Trust) commissioned the programme to take referrals from secondary care. Delivery of the pathway was modelled on the original feasibility programme. With consideration to the limitations identified within the original pathway and changes to the funding model, several logistical and organisational features were revised for the new replication programme. To increase the generalisability of quality improvement research, it is important to evaluate the changes implemented within a replication programme.^19^ Hence, this report evaluates the effectiveness of the quality improvement changes implemented in the adapted CHAIN programme, which was introduced in February 2018. A quality improvement framework has been used to discuss the outcomes of the intervention and its internal and external validities.^19^

## Methods

This is a quality improvement study, reported in accordance with the Standards for Quality Improvement Reporting Excellence (SQUIRE) 2.0 guidelines^20^ and with consideration of published guidance on how to increase the generalisability of quality improvement research.^19^ SQUIRE is intended for reporting the range of methods used to improve healthcare and provides common ground to share these discoveries. The NHS Health Research Authority tool^21^ and Research Department at The Royal Bournemouth Hospital confirmed that ethical approval was not required as this is a quality improvement study. In keeping with good practice, the ethical principles for medical research outlined in the Declaration of Helsinki^22^ were followed.

## Service delivery

The replicated CHAIN programme was delivered at a community-based leisure centre, close to the funding hospital, with good access to public transport and parking. While the original course was run in the evening, the adapted pathway was offered at both lunchtime and in the evenings to suit both the retired and working participant. The referral process was refined so that all participants were referred from the orthopaedic outpatient department of the funding hospital. Previously, patients were referred through their general practitioner (GP). Given that all referrals to the replication programme were from secondary care, it was anticipated that some participants would be less willing to take part due to pre-existing beliefs surrounding their required treatment and the consequences of exercise participation.^23^ In addition, as all referrals were direct from an orthopaedic outpatient department in secondary care, it was predicted that participants may have more severe osteoarthritis, and so may have worse symptoms, such as pain, function or stiffness,^24^ when compared to the original cohort.^15^

## Eligibility

All potential participants were assessed for eligibility by either an orthopaedic consultant or a member of the consultant surgeon’s team (which consisted of experienced lower limb surgeons, completing a hip fellowship and advanced practice physiotherapists). Clear inclusion and exclusion criteria were provided on a clinician referral form, to ensure those who were referred were appropriate for the course. Patients were considered suitable to take part if they had symptomatic hip osteoarthritis. Exclusion criteria were consistent with the local GP exercise referral guidelines and included unstable angina, poorly controlled heart failure, new or uncontrolled arrhythmias, resting or uncontrolled tachycardia (resting heart rate >100 bpm), resting systolic blood pressure >180, resting diastolic blood pressure >100, high levels of frailty, significant functional limitations, body mass index (BMI) 40+ and febrile illness. A patient referral form was created so that participants could consent to having their information shared between the referring hospital, the leisure centre delivering CHAIN and the university evaluating the programme. In addition, a patient information leaflet was provided at the point of referral to clearly outline the service offered and the next steps in the referral process. The referral forms and patient information leaflet were made freely available online so that the process was transparent to both clinicians and patients.

## Intervention

Consistent with the original pathway, participants presenting with hip osteoarthritis participated in 30 min of education (delivered by a senior physiotherapist) and 30 min of static cycling (led by an exercise instructor) once a week for 6 weeks. Education topics reflected the NICE guidelines and included themes such as the benefits of exercise, cycling technique, management strategies for osteoarthritis, complementary therapies, pain relief and assistive devices. The static cycling sessions were designed with an entry level class at week 1 and progressively increased in intensity so that by week 6 the intensity was equivalent to that of a standard static cycling session (such as that offered at the leisure centre). The first week consisted predominately of low-intensity cycling with three short blocks of speed and resistance work. These blocks were progressed, so that by week 6, participants were completing 30 min of high-intensity speed and resistance exercise. Participants were provided with a home exercise programme and an exercise diary to complete in their own time. The home exercise programme included variations of different stretching exercises, for example, calf raises, mini lunges, mini squats and hip flexor stretches.

## Measures

In the 2 weeks preceding the start of the course, participants attended a one-to-one baseline assessment with an exercise referral specialist from the leisure centre where the programme was delivered. Participants were introduced to the course, asked about their relevant medical history and completed an assessment including functional tests and validated health-related questionnaires. Data collection was primarily quantitative-based, in addition to three open-ended questions used to evaluate the course. While some outcome measures remained consistent with the original pathway (Timed Up and Go (TUG),^25^ Oxford Hip Score,^26^ Hip Osteoarthritis Outcome Score (HOOS) function,^27^ EQ5D VAS, EQ5D-5L Utility,^28^ pain at rest and on weight bearing), several changes were made to the replicated programme. The five times sit–stand test was substituted by the 30-s chair stand test, as recommended by the Osteoarthritis Research Society International (OARSI) Society for patients with hip or knee osteoarthritis.^25^ The Harris Hip score^29^ was removed as it was originally developed for assessment follow hip surgery and the non-arthritic hip score^30^ was considered unnecessary given the use of the HOOS questionnaire. Participants were asked to set three personal goals they would like to achieve by the end of the programme, report any worries or concerns they had regarding their participation and any perceived barriers to taking part. Subsequent to completing the cycling and education intervention, participants attended a follow-up appointment with the same exercise referral specialist. They repeated their functional tests and questionnaires and were asked to review the goals they set at the start of the programme. Participants were asked what they had found useful about the course, what they would like to see improved in the future and whether they would recommend the programme to a friend.

To reduce the likelihood of incomplete or inaccurate data sets and to increase efficiencies within the CHAIN programme, the data collection process was changed from paper-based to a web-based system (software providers: Actipath, Bournemouth, UK (http://actipath.co.uk/)). The secure, online platform was developed to provide end-to-end management of participants throughout the course and included real-time outcome analysis and participant communication via email and text. It ensures consistency with outcome data collection and delivery of session content and is therefore important for the future scalability of the CHAIN programme. Two previously reported limitations of the CHAIN programme were as follows: (1) variations in the amount of cycling completed outside of CHAIN and (2) poor compliance with the home exercise programme.^15^ At an attempt to reduce these limitations, a feature was added to the system whereby an email or text was sent to each participant at the end of the CHAIN session. The communication included the home exercise programme, an educational video and a 30-min indoor cycling tutorial to match that of the cycling session completed that week. The system was also used to generate progress reports, which were sent to the participant, their GP and the referring hospital once the follow-up assessment was complete. The software also allowed the funding hospital to monitor the 18-week referral to treatment pledge, whereby the NHS aim to provide non-urgent treatments to patients within 18 weeks of referral.

## Analysis

This evaluation includes all referrals to the replicated CHAIN programme between February 2018 and September 2019. Two-sided paired *T* tests were used to investigate the changes from pre-programme to post-programme for the Oxford Hip Score, 30-s chair stand test, TUG, pain at rest and on weight bearing, EQ5D health rating and EQ5D-5L Utility score and HOOS function and pain scores. For the 30-s chair stand and TUG tests, scores of zero were considered data errors and were therefore omitted from the final analysis. All data were analysed using IBM SPSS Statistics version 26 (SPSS Inc., Chicago, IL, USA), with the significance level set at *p* < 0.05.

Data from the open-ended questions asking participants to detail what was useful about coming on the programme, what could be improved for future participants and whether they would recommend the programme to others were thematically analysed using an inductive approach to identify key themes. First- and second-order themes were independently identified by two researchers (L.C.B. and T.I.) and any discrepancies between findings were resolved and refined through discussion with the research team. To assess the impact of the adapted course, outcomes from both the original and replication programme are compared in this evaluation.

## Results

**Figure 1.**
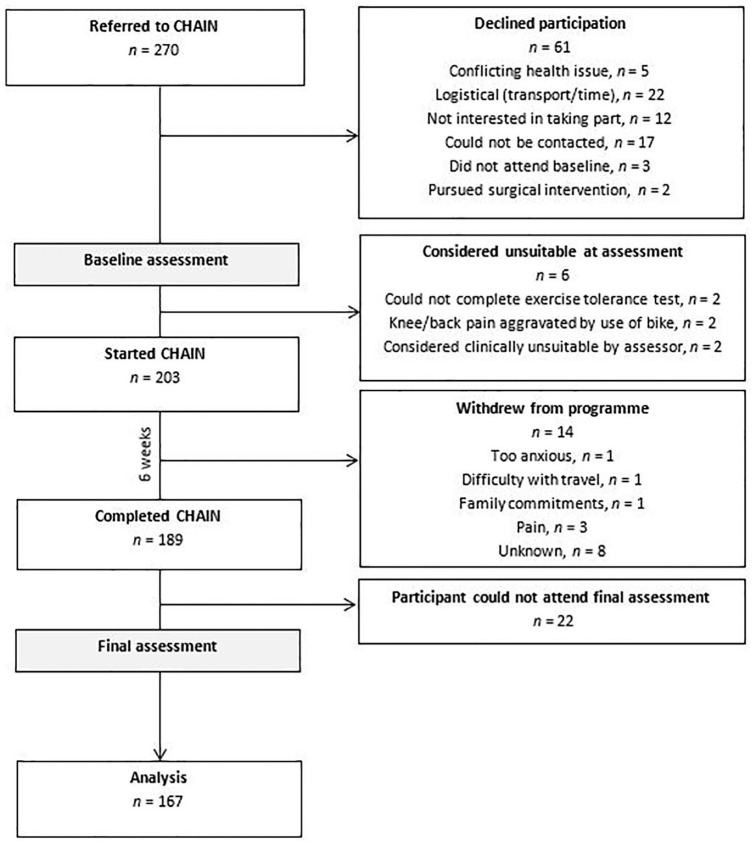
Participant flow chart.

A total of 270 patients were referred to the CHAIN programme between February 2018 and September 2019 (Table 1). In all, 189 completed the programme, and 167 attended their final assessment (98 (59%) female, 69 (41%) male). The mean baseline Oxford Hip Score was 29.17 and 141 (84%) participants had a diagnosis of hip osteoarthritis. The mean age of the participants was 61.98 years. Figure 1 shows the flow of participants through the programme. In all, 61 participants declined participation or could not be contacted. Reasons for declined participation included conflicting health issues (5), a lack of available transport or time (22), no interest in taking part (12) and the decision to pursue surgical intervention (2). Average class attendance was five out of six sessions.

**Table 1.** Participant characteristics, values are mean (SD).

Outcome	*n* (%)
Gender, *n* (%)	
Female	98 (59%)
Male	69 (41%)
Age (years)	61.98 (11.23)
Hip involved, *n* (%)	
Both	40 (24%)
Left	47 (28%)
Right	80 (48%)
Primary diagnosis, *n* (%)	
Osteoarthritis	141 (84%)
Post-traumatic	5 (3%)
Rheumatoid arthritis	4 (2%)
Avascular necrosis	1 (1%)
Other	16 (10%)
Weight (kg)	
Pre-programme	80.40 (17.65)
Post-programme	79.98 (17.52)
Height (cm)	170.11 (9.56)
Body mass index (BMI)	
Pre-programme	27.67 (4.86)
Post-programme	27.52 (4.81)
Baseline Oxford Hip Score	29.17 (6.89)

Six participants were considered unsuitable for the programme by the exercise referral specialist at their baseline assessment. Of the 201 participants who started the 6-week intervention of cycling and education, 14 withdrew from the programme (too anxious to participate (1), difficulty with travel (1), family commitments (1), in too much pain (3), unknown reasons (8)). In all, 21 participants completed the cycling and education intervention but did not attend their follow-up appointment. This analysis includes 167 participants who completed the whole course (Table 1).

The participants on the replicated CHAIN programme did not differ from the original cohort^15^ in terms of mean age (original pathway: 62.23 ± 9.27 years, adapted pathway: 61.98 ± 11.23 years, *p* = 0.85) or pre-programme weight (BMI) (original pathway: 27.72 ± 4.61, adapted pathway: 27.67 ± 4.86, *p* = 0.93), however, presented with worse hip symptoms at baseline as measured by the Oxford Hip Score (original pathway: 33.07 ± 8.18, adapted pathway: 29.17 ± 6.89, *p* < 0.001). Consistent with the findings from the original pathway, participants made significant improvements to Oxford Hip Score (mean change: 4.75, 95% confidence interval (CI): 3.74–5.76, *p* < 0.001); 30-s chair stand score (mean change: 1.81, 95% CI: 1.38–2.25, *p* < 0.001); TUG score (mean change: 0.92, 95% CI: 0.64–1.19, *p* < 0.001); HOOS function (mean change: 9.81, 95% CI: 7.52–12.11, *p* < 0.001); HOOS pain (mean change: 8.94, 95% CI: 6.63–11.24, *p* < 0.001); EQ5D health rating (mean change: 4.33, 95% CI: 1.50–7.16, *p* < 0.001); EQ5D-5L score (mean change: 0.06, 95% CI: 0.03–0.09, *p* < 0.001); pain at rest (mean change: 1.02, 95% CI: 0.61–1.45, *p* < 0.001) and pain on weight bearing (mean change: 1.11, 95% CI: 0.66–1.75, *p* < 0.001) (Table 2).

**Table 2.** Pre- and post-CHAIN mean outcome scores.

Outcome measure	*n*	Mean pre-score (SD)	Mean post-score (SD)	Mean health gain (95% CI of pre–post improvement)	SD	Sig. (two-tailed)
30-s chair stand (*n*)	166	12.33 (4.58)	14.14 (5.40)	1.81 (1.38–2.25)	2.85	<0.001
Timed Up and Go (s)	165	8.15 (3.17)	7.24 (2.50)	0.92 (0.64–1.19)	1.79	<0.001
Oxford Hip Score	167	29.17 (6.89)	33.93 (8.22)	4.75 (3.74–5.76)	6.62	<0.001
Pain at rest (VAS)	167	3.33 (2.59)	2.31 (2.41)	1.02 (0.61–1.45)	2.77	<0.001
Pain on weight bearing (VAS)	167	4.32 (2.74)	3.20 (2.65)	1.11 (0.66–1.57)	2.96	<0.001
EQ5D health rating	167	65.03 (17.21)	69.37 (18.41)	4.33 (1.50–7.16)	18.53	0.003
HOOS ADL	167	62.86 (16.40)	72.68 (18.54)	9.81 (7.52–12.11)	15.03	<0.001
HOOS pain	167	60.53 (16.20)	69.46 (17.53)	8.94 (6.63–11.24)	15.1	<0.001
EQ5D-5L score	167	0.63 (0.16)	0.68 (0.21)	0.06 (0.03–0.09)	0.17	<0.001

CHAIN: Cycling against Hip Pain; SD: standard deviation; CI: confidence interval; HOOS: Hip Osteoarthritis Outcome Score; VAS: visual analogue scale; ADL: activities daily living.

Oxford Hip Score: scored 0–48 with a score of 40+ suggesting satisfactory function. 30-s chair stand: number of times participant rises and sits from a chair in 30 s. Timed Up and Go test: the time taken (in seconds) to rise from a chair, walk 3 m, then turn and sit back on the chair. Pain at rest and pain on weight bearing: scored 0–10 with 0 being no pain and 10 being the worst imaginable pain. EQ5D Health Rating: The number between 1 and 100 that the participant considers their health to be on that day (1 = worst, 100 = best). EQ5D-5L score: standardised tool used to measure generic health, index score calculated from the UK value set provided by EQ5D.^31^ HOOS function and pain: score out of 100 concerning self-reported physical function/pain with 100 indicating no problems and 0 extreme problems.

Reponses to the questions asked on the three most useful elements of the course were divided into three main themes: symptom benefits, knowledge benefits and motivation/confidence benefits. Symptom benefits included reports of reduced pain or improved fitness, strength, sleep or mobility. Knowledge benefits mostly incorporated comments on increased knowledge on how to self-manage arthritic symptoms following the education session and improved cycling technique. The final theme was applied to comments related to greater confidence while cycling or an increase in motivation to exercise.

These results are similar to the findings from a different cohort of patients within the original feasibility programme, whereby participants spoke of the social and physical benefits of exercise in a group, an increased confidence and motivation to exercise and knowledge on pain management.^15^ Engagement figures for the education and cycling tutorials provided online suggested participants remained motivated to learn and exercise in their own time. Importantly, 166 of 167 (99%) participants reported that they would recommend the course to a friend, and 1 participant said they would consider recommending it depending on the longer term results. These results are comparable to the original programme, whereby 100% of participants reported that they would recommend the course.^15^

When asked what could be improved about the course, 107 participants responded with at least one suggestion. In all, 50 (35%) suggestions were made regarding the education session (e.g. additional topic areas, longer group discussion), 42 (29%) were related to the course structure (e.g. longer duration of course, delivered at different locations), 18 (13%) were related to the cycling session (volume of music or comfort of equipment), 12 (8%) asked for more support in the longer term (e.g. an additional follow-up session, specialised ‘post-CHAIN’ spin classes) and 10 (7%) suggestions were with regard to the home exercise programme (e.g. increased clarity on dose, demonstrations of complicated stretches). These suggestions were similar to findings from a Patient and Public Involvement (PPI) forum conducted after the original course, whereby participants suggested a longer course and more time for group discussion.^32^

## Discussion

The findings from this quality improvement study suggest that people with hip osteoarthritis, referred for conservative treatment from an orthopaedic outpatient clinic in secondary care, can benefit from a 6-week cycling and education intervention. Participants improved in terms of function, pain and patient-reported quality of life. The changes in HOOS function, pain and TUG are likely to be clinically significant, based upon previously reported minimally clinically important improvement (MCII) in the hip osteoarthritis population.^33–35^ The change of 1.8 in the 30-s chair stand score did not quite reach the MCII of two to three chair stands,^33^ and likewise, the change of 0.06 in EQ5D-5L score was just below the threshold 0.07 reported in non-surgical hip and knee osteoarthritis patients.^36^ Similarly, the Oxford Hip Score change of 4.75 fell below the MCII of 7 points reported by Martin-Fernandez et al.^37^

While the participants on the original^15^ and replicated CHAIN courses did not differ in terms of age or pre-programme BMI, the second cohort presented with worse hip symptoms at baseline, as measured by the Oxford Hip Score.^26^ Nonetheless, participants made similar improvements in terms of clinical and patient-reported outcome measures over the 6-week period. These findings increase the external validity and generalisability of the CHAIN programme as a conservative treatment pathway, given the similarity in results from participants referred from both primary and secondary care. In addition, our results support the efficacy of the quality improvement changes implemented within the replicated CHAIN programme. While these findings are specific to the local context, they are important for the future scalability and dissemination of the CHAIN programme.

Our results are perhaps not surprising given that exercise is recommended for the management of osteoarthritis irrespective of pain, functional status and disease severity.^9,38,39^ The approach of combining exercises to increase strength, flexibility and aerobic capacity is reported to be the most effective for lower limb osteoarthritis,^40^ and aerobic exercise is considered the most beneficial for pain and objective performance (e.g. walking speed, strength, range of motion).^12^ Cycling can induce muscle hypertrophy and increase aerobic capacity for older adults^41^ and has been reported to enhance balance and proprioception.^42^ The continuous cycling motion requires repetitive end-range joint mobilisation that may help to reduce pain by mechanisms such as inhibiting reflex muscle contraction, reducing intra-articular pressure and the level of joint afferent activity. In addition, the cardiovascular benefits of cycling are important given the higher risk cardiovascular risk in this patient group^43^ and the increased risk of cardiovascular excess death with longer duration of hip and knee osteoarthritis.^44^

While our findings are promising, further work is needed to increase participation in exercise for adults with osteoarthritis. Of the 270 participants referred onto the replicated CHAIN programme between February 2018 and June 2019, 167 (62%) completed the course and attended their follow-up appointment. While some cases of withdrawal from the course were due to medical necessity, it was not uncommon for participants to decline participation or fail to attend their baseline or completion assessment. The original course saw 96 of the 114 (81%) referrals complete the course.^15^ As anticipated, the disparity between dropout rates for the two courses may have been due to the revised referral pathway. As all referrals were direct from secondary care, where patients may have wanted surgical intervention, it was predicted that they may be less willing to participate and have worse symptoms, such as pain, function or stiffness. While recent evidence has led to the re-evaluation of physical exercise as a therapeutic modality for people with osteoarthritis, traditional beliefs were that exercise or physical overuse could play a role in the pathogenesis of the disease.^45^ Attitudes and behaviours can be shaped by beliefs about chronic hip pain, and as a consequence, those who are confused and unsure of how to manage their symptoms may avoid activity due to fear of causing harm.^46^ Reasons given for declined participation were similar to previously reported barriers to exercise for people with osteoarthritis, described within the biopsychosocial model of health.^47^ Influencing factors have been presented under the three conceptual domains of physical health (e.g. level of pain), intrapersonal factors (such as motivation) and social-environmental factors (such as transport or facilities).^23,47,48^ While the addition of the lunchtime session to the replicated CHAIN programme was popular with many older adults who did not like travelling in the dark, access was still an issue given the number of people who declined participation due to transportation and time.

The findings from this study are comparable to the results of published larger-scale evaluations of exercise interventions for the treatment of hip osteoarthritis, such as Good Life with osteoArthritis in Denmark (GLA:D^®^)^49^ and the Escape-pain programme.^50^ GLA:D includes 12 sessions of supervised neuromuscular exercise in addition to 3 education sessions, and Escape-pain offers 10 sessions which include education, strengthening and resistance exercises, cycling and functional and balance exercises. A feasibility study of GLA:D in Canada (*n* = 22) reported a mean increase of 8.8 (95% CI: 4.7–12.9) in HOOS pain scores and a mean increase of 12.0 (95% CI: 6.3–17.8) in HOOS function scores.^51^ These changes are greater than those found in this study; however, this may be explained by GLA:D including an additional six sessions of exercise. A feasibility trial of the Escape-pain initiative (*n* = 48) also found moderate improvements in HOOS pain (effect size: 0.5) and HOOS function (effect size: 0.4).^50^ To further validate the CHAIN programme, funding has been secured to compare the CHAIN programme to standard physiotherapy care in a randomised controlled trial (study ID: ISRCTN19778222).

## Limitations

This study was designed to evaluate the effects of a replicated quality improvement study. Nonetheless, a lack of control group may result in an overestimation of the treatment effects when compared to the effects found in controlled clinical trials. Funding has been secured to compare the CHAIN programme to standard physiotherapy care in a randomised controlled trial, where recruitment bias can be controlled. In addition, while we utilised the Oxford Hip Score to report osteoarthritis symptoms, we did not collect or report the severity of hip osteoarthritis through radiographic grading.

## Conclusion

A 6-week cycling and education intervention for the treatment of hip osteoarthritis can provide benefits to function, pain and quality of life for patients referred from secondary care. In addition, participants reported an increase in knowledge, confidence and motivation to exercise. These results are consistent with findings from patients who were referred from primary care and further support the potential of the pathway in the conservative management of hip osteoarthritis.
